# Motivational Understanding of MOOC Learning: The Impacts of Technology Fit and Subjective Norms

**DOI:** 10.3390/bs13020098

**Published:** 2023-01-23

**Authors:** Lingfeng Dong, Ting Ji, Jie Zhang

**Affiliations:** 1 Alibaba Business College, Hangzhou Normal University, Hangzhou 311121, China; 2 School of Management, Zhejiang University, Hangzhou 310058, China; 3 School of Business, Hangzhou City University, Hangzhou 310015, China

**Keywords:** MOOCs, intrinsic motivation, extrinsic motivation, subjective norm, technology fit

## Abstract

This study examines the mechanisms underlying the relationship between motivation and massive open online course (MOOC) learning intention, and the contextual moderators that affect this mechanism. Drawing on motivational theory and the related literature, this study investigates how motivation affects students’ intention to learn with MOOCs and the related meditation and moderation effects. The findings of our study show that both extrinsic and intrinsic motivations have a significant influence on intention to learn with MOOCs. In addition, the results also suggest that the relationship between extrinsic motivation and intention to learn is mediated by the technology fit perceived by learners but not the relationship between intrinsic motivation and intention to learn. Furthermore, the relationships between both extrinsic and intrinsic motivation and intention to learn are moderated by subjective norms. This study enriches the extant literature on the impact of technology fit and subjective norms on MOOC learning. Few studies have focused on how students can be effectively encouraged to take MOOCs. Given the lack of theoretical and empirical research, this study developed a theoretical model and conducted an empirical study to fill the research gap.

## 1. Introduction

The rapid development of information technology offers new educational opportunities. One of the most significant innovations is the development of massive open online courses (MOOCs) [1,2,3]. MOOCs have various advantages over traditional in-class courses, such as accessibility (i.e., anyone can access this type of online course), reduced time restrictions (i.e., students can learn with MOOCs whenever they are convenient), and wide discussions (i.e., learners can discuss issues with others worldwide). Realizing the great potential at different levels of education, researchers have widely explored various issues related to why and how people come to MOOCs and what factors contribute to learners’ MOOC performance, such as teachers’ motivation to prepare them [4,5], the effectiveness of different types of MOOCs [6,7], and students’ attitudes toward MOOCs [8,9].

Our comprehensive review indicates that most of the studies focused on improving students’ MOOC learning intention and sustained participation learning behavior from the aspects of technology application, external reward, and course design. For example, the practical means used by both platforms and teachers to encourage students to take MOOCs are certificates and credits [10,11]. That is, platforms promise students that they will earn a certificate if they pass specific MOOCs, while teachers try to encourage students to take MOOCs by offering credits as an incentive. Though the findings of these studies provide rich knowledge in this direction, significant problems, such as low rates of enrolment, retention, and completion, remain widespread [12,13,14], and little is known concerning how students can be effectively encouraged to take MOOCs. Given that motivation is important in sustaining people to engage in an activity and achieve results, it is imperative to call for research examining students’ motivation to take MOOCs [15].

Towards this direction and based on motivational theory and theory of planned behavior, our research examined the mechanisms underlying the relationship between extrinsic and intrinsic motivations and students’ intention to learn with MOOCs, as well as the contextual moderators that affect this mechanism. We propose that extrinsic and intrinsic motivations significantly affect a students’ intention to learn with MOOCs, but their underlying effects on behavioral intention could differ. We used a survey method to test this proposition. The findings of this study make the following theoretical contributions. First, despite recent discussions regarding possible means to improve students’ engagement with MOOCs [15,16], investigations emphasizing students’ motivation to learn with MOOCs are rare. As one of the initial attempts to pursue this research direction, this study examined whether and how students’ extrinsic and intrinsic motivations influence their intention to learn with MOOCs. Second, the findings of this study provide insight into how students’ assessment of technology fit affects the relationships between different types of motivation and intention to learn with MOOCs. The relationship between extrinsic motivation and behavioral intention was found to be fully mediated by students’ evaluation of technology fit, which calls for researchers to consider the differences between extrinsic and intrinsic motivations. Third, the findings of this study also offer contributions by identifying moderator variables (i.e., subjective norms) that affect the relationships between both types of motivation and intention to learn with MOOCs.

## 2. Theoretical Background and Hypotheses

### 2.1. Massive Open Online Courses (MOOCs)

Currently, with the rapid development of technology, the world is dramatically changing in wide areas, and the education industry is no exception. One of the greatest innovations is the MOOC. MOOCs are an educational model based on internet technology. Unlike traditional courses, where teachers and students mainly discuss a subject within a stipulated time period and at a fixed location, MOOCs provide freedom in both areas [1]. As long as they have internet access, learners worldwide can log in to a specific platform to choose from a broad range of courses anytime and anywhere [11]. This innovative educational model also has a range of other advantages: large variety of courses available, large number of courses delivered by professors from world-renowned universities, easy comparison of courses on the same subject delivered by different teachers who may provide knowledge supplementing each other, etc. [2,4]. These broad advantages are driving the fast development of MOOCs, making it one of the most popular educational models in the world. 

Despite these great merits, teaching practitioners and educational researchers are concerned about a range of issues relating to the launch of such an educational model. For instance, scholars have widely argued that the preparation of MOOCs is time- and energy-consuming, much more so than in traditional teaching [17]. One noteworthy case is that of Roger Barr, who reported that to deliver a course, he and his staff did more than 620 h of preparatory work and spent 420 h on course delivery, and that others, such as teaching assistants and providers of instructional and technical support, performed at least 200 h of work [18]. Likewise, Robert Sedgewick spent hundreds of hours preparing related materials, and devoted 14 days to recording each lecture [19]. This educational model also involves one teacher facing hundreds of students simultaneously, which may significantly hinder communication between teacher and students [20]. Although these problems are acknowledged as significant issues, an even more urgent and severe issue is how to attract and better encourage students to learn with MOOCs and retain their knowledge [21]. 

Several practical attempts have been made to encourage students to learn with MOOCs. For instance, some MOOC providers offer completion certificates to attract students [11]. Although this is recognized as an effective way to stimulate students, students are widely concerned about the usefulness of such a certificate, because it is not fully acknowledged by employers and educational institutions [15]. From teachers’ perspective, realizing that the knowledge gained from in-class discussions is not sufficient for students [22], teachers encourage students to learn with MOOCs as a supplement. Offering students course credits is a feasible way to encourage them to learn with MOOCs [6]. However, in a survey of 103 professors, around 72% argued that students do not qualify for the credits even if they finish courses with MOOCs [19]. 

**Figure 1:**
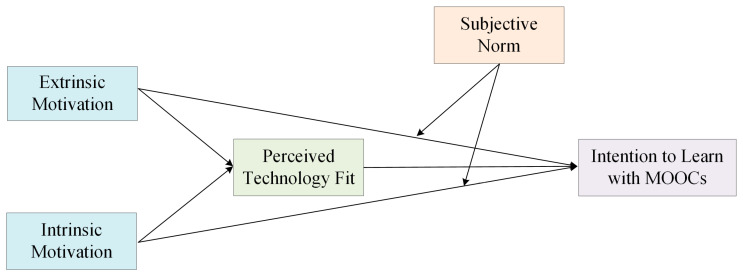
Research model.

Though the value of the strategy referred above (i.e., incentivization with certificates or credits) has been widely acknowledged, researchers still argue that they cannot go beyond a superficial level of understanding students, because people’s behavior, most of the time, can be motivated by both the extrinsic and intrinsic motivations [23,24]. Very recently, a few studies have begun to pay attention to ways of intrinsically encouraging students to engage with MOOC learning [25,26]. Barak, et al. [25] examined the effects of related antecedents (i.e., language and social engagement) that influence students’ motivation, but little is known concerning broader influential factors. Likewise, the effects of intrinsic and extrinsic motivations on students’ behavior are unclear. Accordingly, based on this research gap, this study sought to further understand why and how different dimensions of motivation (i.e., intrinsic and extrinsic motivations) impact students’ MOOC learning behavior and the related mediation and moderation effects. In doing so, we drew on motivational theory, technology fit, and subjective norm-related literature. Figure 1 presents the research model. 

### 2.2. Motivational Theory

Motivational theory is used to explain people’s motivation to do something. Considering the nature of human beings, the theory introduces two types of motivation: intrinsic motivation and extrinsic motivation [27]. People are intrinsically motivated to do something because they are really interested, or find it enjoyable, and are engaged in the task for its own sake; people are extrinsically motivated to do something because of focal intentions such as obtaining external rewards [28]. In line with the central thesis of this theory, we conceptualized students’ intention to learn with MOOCs as motivated by both extrinsic and intrinsic motivations. As noted above, some MOOC platforms and teaching practitioners have begun to encourage students by using certificates and credits as incentives, which is consistent with the conceptualization of extrinsic motivation [10,11]. 

Intrinsic motivation, in this specific context, refers to students finding an MOOC course enjoyable and interesting. There are two dimensions of an MOOC course: content and its representation. With regard to the MOOC content, students may appreciate a course because they cannot obtain the content from offline teachers or because offline teachers do not perform as well as MOOC teachers. With respect to MOOC representation, students may enjoy an MOOC course even if the same content is available offline. In this study, we conceptualized students’ intrinsic motivation to take an MOOC course from a cohesive perspective, including either or both of these two dimensions. 

Previous studies have argued from different perspectives that extrinsic motivation can have a significant influence on intrinsic motivation [29,30,31,32], while others have asserted that extrinsic motivation hinders people’s intrinsic motivation [33,34] and, thus, the literature presents inconsistent findings. Although it is still not clear how extrinsic motivation affects intrinsic motivation (i.e., enhancing or undermining), based on the central thesis of motivational theory, researchers agree that both extrinsic and intrinsic motivations can lead to an intention to do something [35]. We conjecture that both of these dimensions could lead to positive behavioral intentions.

**Hypothesis 1** **(H1).** *Extrinsic motivation positively influences students’ MOOC learning intention*.

**Hypothesis 2** **(H2).** *Intrinsic motivation positively influences students’ MOOC learning intention*.

Based on a basic understanding of the effect of students’ extrinsic and intrinsic motivations on their behavioral intention to learn with MOOCs, two contextual factors must be considered. One is closely related to the MOOC technology itself, while the other concerns how environmental factors (e.g., subjective norms) influence students’ MOOC learning. In the following sections, we discuss how these two factors, technology fit and subjective norms, affect students’ intention to learn with MOOCs. 

### 2.3. Perceived Technology Fit

As noted, the MOOC is an innovative internet-based educational model. Before the emergence of MOOCs, students primarily obtained knowledge offline through face-to-face discussions with teachers and peers or by researching materials in the library [1]. The MOOC has unique characteristics that differentiate it from traditional learning: students not only learn by themselves online, with less opportunity to discuss problems at once, but also, most of the time, share one teacher with thousands of other students worldwide [36]. This could easily lead students to worry about the fit of a technology in providing effective learning. Research in a wide range of disciplines, such as education [37] and information systems [38,39], have demonstrated the key role of “technology fit” in influencing final outcomes. However, there is little investigation of whether fit matters in the MOOC context. 

Technology fit is an essential part of the learning support service system, which can help people to effectively carry out and promote online learning by providing learners with information, resources, technology, and emotional and management support [37]. In e-learning, due to the separation of teachers and students and the diversity of learners, personalized learning support, especially the matching of learner information and technology-based resources, will significantly impact on students’ MOOC learning intention [40,41]. Therefore, when a student is extrinsically motivated by external rewards to learn with MOOCs, if he/she faces the problem of a lack of fit between the course and MOOC technology (i.e., the MOOC is not a good way to learn a subject), he/she typically will not choose to learn with MOOCs. Meanwhile, if a student finds that the MOOC is not an appropriate way to learn, even if he/she thinks that MOOC learning is enjoyable and interesting, it is also possible to stop. Therefore, we propose that the effects of both extrinsic and intrinsic motivations on student MOOC learning are mediated by assessments of technology fit.

**Hypothesis 3** **(H3).** *Perceived technology fit mediates the relationship between extrinsic motivation and MOOC learning intention.*

**Hypothesis 4** **(H4).** *Perceived technology fit mediates the relationship between intrinsic motivation and MOOC learning intention.*

### 2.4. Subjective Norm

As discussed above, we hypothesized that students’ intention to learn with MOOCs is affected by their own motivations and technology fit. In addition to these two factors, students’ learning intention is also influenced by subjective norms [42]. Drawing on the theory of planned behavior, behavioral intention is influenced by attitudes and subjective norms and is a direct factor that determines individual behavior [43]. According to Dong, et al. [44], Ajzen [45], subjective norms are defined as the social pressure an individual perceives when deciding whether to perform a certain behavior. It reflects the influence of important individuals or groups on people’s behavioral decision making. For example, students’ MOOC learning attention can be influenced by their social environment, that is, by the behavior of their classmates, friends, and teachers [46,47]. Subjective norms are influenced by normative belief and motivations to comply. More specifically, normative belief refers to the degree to which one believes that people who bear pressure on one’s actions expect one to perform the behavior, and motivation to comply refers to the degree of one’s compliance with each of one’s referents. 

We conjecture that subjective norms influence the relationships between extrinsic and intrinsic motivations and behavioral intentions differently because these two underlying relationships are different in nature. Specifically, extrinsic motivation focuses on the goal-driven reasons and is stimulated by external stimuli, such as certificates or other types of reward. It can be easily affected by others’ behavior (e.g., classmates’ choices or friends’ suggestions). On the other hand, intrinsic motivation is generated from learners’ personal interests and enjoyment, which are relatively sustainable and stable. Individuals endorse intrinsic motivation when they engage in activities for personal value goals and to attain self-validation results. 

Subjective norms represent the perceived social pressure from significant others to engage in the target behavior [44]. Therefore, when learners have a high degree of subjective norms, they are more likely to consciously or unconsciously form or change their attitude and behavior according to certain group norms or opinions of the majority. At this point, if they are driven by external motivation to participate in MOOC learning, not out of interest in the course itself, but to obtain some separable result, such as a credit or reward, they are more likely to be influenced by the external environment, thus increasing their intention to study in MOOCs.

In contrast, if students who participate in MOOCs are motivated by intrinsic motivation, such as interest, achievement goals, and value beliefs, a high level of subjective norms may reduce their MOOC learning intention. According to the over-justification effect, external rewards or stimulation may decrease intrinsically reinforced behaviors [48,49,50]. Specifically, learners originally participate in MOOC learning for internal reasons, such as interests or hobbies. However, when they feel pressure or expectations from the surrounding environment or groups, the reasons for behavior are “excessive”, prompting people to interpret their behavior as external reasons while ignoring the original internal reasons. Once the external stimulus ceases, people’s behavior intention tends to stop for lack of reason [33,34,51,52]. Therefore, we propose the following hypotheses:

**Hypothesis 5** **(H5).** *Subjective norms moderate the relationship between extrinsic motivation and students’ MOOC learning intention, such that the relationship is positive and stronger when subjective norms are high rather than low.*

**Hypothesis 6** **(H6).** *Subjective norms moderate the relationship between intrinsic motivation and students’ MOOC learning intention, such that the relationship is positive and weaker when subjective norms are high rather than low.*

## 3. Research Methodology

We conducted a survey study by considering the generalizability. To enhance the validity, we adapted relevant measurement items from previous works. Table 1 presents the related measurement items and sources. All questions were designed to be answered on a seven-point scale anchored from “strongly disagree” to “strongly agree”. 

### 3.1. Data Collection

In the survey, we emphasized college student samples. Various forms of the questionnaire could be chosen for convenience, including a paper-based questionnaire that students could complete by hand; a paper-printed website link that students could complete on a PC by typing the link in a browser; and an online questionnaire that students could access by scanning a paper-based QR code with a smartphone. Every student in each class was promised one additional mark on their final exam if they completed the questionnaire. Students were required to ask questions during the data collection process if they had concerns. Before distributing the questionnaire, the classroom teachers had been trained to answer all questions their students might have. The data were collected over one week from different classes in different universities. Ultimately, 277 questionnaires were collected. After twenty-nine were rejected as incomplete (i.e., some were not finished, with questions unanswered; some students had responded “strongly disagree” to all of the questions), we kept 248 questionnaires for data analysis. The average age of the respondents was 21.32, and 61.29% were male. The samples covered more than eight different colleges, including the schools of management, economics, computer science, law, education, and electronic engineering. Among them, 52.02% were freshmen, and most of the rest were sophomore and junior students, except for 5 seniors. 

### 3.2. Reliability and Validity

Before testing the hypotheses, we assessed the reliability and validity of the constructs. In the data analysis, statistical tests were carried out at the 5% level of significance. The reliability of the constructs was assessed by the Cronbach’s alpha. Values larger than 0.70 indicated good reliability. The Cronbach’s alpha of the constructs was as follows: extrinsic motivation (0.913), intrinsic motivation (0.861), technology fit (0.961), subjective norms (0.860), and intention to learn with MOOCs (0.947). According to Fornell and Larcker [56], all constructs had adequate reliability. 

The construct validity was tested using factor analysis with principal component analysis and varimax rotation. To ensure rigorous analysis, two dimensions of validity must be tested, namely, convergent and discriminant validity. Convergent validity should be tested to assess whether items within the same construct highly correlate amongst themselves; discriminant validity, on the other hand, should be assessed by determining whether the items load more highly on their intended construct than on others [57,58]. A loading between 0.45 and 0.54 is generally considered fair; a loading between 0.55 and 0.62 is generally considered good; a loading between 0.63 and 0.70 is generally considered very good; and a loading is considered excellent if it is higher than 0.71 [44]. The factor loading analysis indicated that all constructs in the model had both excellent convergent and discriminant validity (see Table 2). 

### 3.3. Common Method Bias

Considering that all questionnaires were from students’ self-reporting, the common method bias might be a potential issue. To minimize the presence of potential threats, we designed the online survey via the ex-ante approach [59]. The ex post results of Harman’s single-factor test suggested that the total variance explained by a single factor was 33.49%, which is much lower than the commonly accepted threshold [57]. Therefore, the common method bias was not a severe issue.

### 3.4. Hypotheses Testing

**Figure 2:**
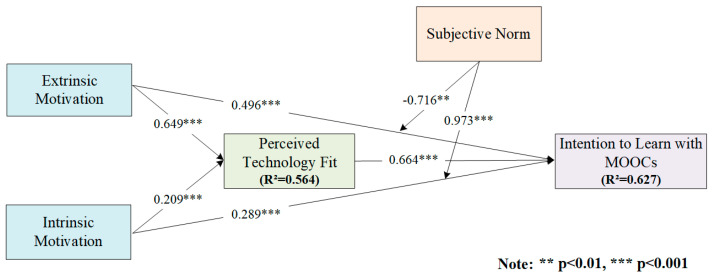
Empirical study results.

We used SmartPLS 3.0 software packages to examine the research model [60]. Specifically, to determine the standardized estimates of the conceptual model, we empirically examined the structural model using the PLS algorithm. In addition, we employed the bootstrapping method to estimate the significance and t-statistics of each hypothesis path within the structural model [61]. Figure 2 presents the empirical study results.

The test of the main effects did not take the mediator or moderator into consideration. We tested how extrinsic and intrinsic motivations affected intention to learn with MOOCs directly. The results show that both extrinsic motivation (β = 0.496, *p* < 0.001) and intrinsic motivation (β = 0.289, *p* < 0.001) significantly influenced students’ intention to learn with MOOCs. Hypothesis 1 and Hypothesis 2 are thus supported. 

When testing the mediation effect, we obeyed the normal rule and used four steps in the analysis [62]: (1) test whether extrinsic motivation and intrinsic motivation are significant with intention to learn with MOOCs (Path A); (2). Test whether the perceived technology fit is significant with intention to learn with MOOCs (Path B); (3) test whether extrinsic motivation and intrinsic motivation are significant with perceived technology fit (Path C); (4) add the direct effects of extrinsic motivation and intrinsic motivation on intention to learn with MOOCs and test the significance (Path D). As shown in Table 3, we concluded that the perceived technology fit played a fully mediated role in the relationship between extrinsic motivation and intention to learn with MOOCs. Thus, Hypothesis 3 is supported. In addition, we found that perceived technology fit played a partial mediated role in the relationship between intrinsic motivation and intention to learn with MOOCs. Thus, Hypothesis 4 is supported.

The interaction effects show that subjective norms significantly affected the relationships between both extrinsic motivation (β = –0.716, *p* < 0.01) and intrinsic motivation (β = 0.973, *p* < 0.001) and intention to learn with MOOCs. Both Hypothesis 5 and Hypothesis 6 are well supported. 

## 4. Discussion

A number of interesting findings were uncovered in this study, especially those concerning the moderation and mediation effects. The findings suggest that both extrinsic and intrinsic motivations have a significant influence on students’ MOOC learning intentions. They also show that students’ perception of technology fit mediated the relationship between extrinsic motivation and learning intention but did not mediate the relationship between intrinsic motivation and learning intention. In other words, students’ intention to learn with MOOCs was motivated by both extrinsic and intrinsic motivations, but motivations aroused by external stimuli (e.g., rewards such as certificates or credits) had effects on learning intentions only when students perceived a technology fit with a course. These findings suggest that, for the students who are motivated by external stimuli, they are more easily influenced by factors such as whether the format of the MOOC fits with the focal course they learn. However, when students are motivated by their own intrinsic motivation to pursue knowledge from the course itself, their learning intention is not likely be impacted by disturbance factors. These findings further remind MOOC practitioners on how to motivate students to learn from an MOOC with their intrinsic motivation, e.g., making it be interesting to learners. 

The findings also reveal that subjective norms moderated the relationships between both extrinsic and intrinsic motivations and intention to learn with MOOCs. Interestingly, these two moderations typically were different. That is, subjective norms had a positive influence on the effect of extrinsic motivation on intention to learn and a negative influence on the effect of intrinsic motivation on intention to learn. In other words, as the force of subjective norms increased, the effect of extrinsic motivation on behavioral intention decreased (although it remained significant). This means that when more peers around a student want to take an MOOC course, he/she is less likely to do so, regardless of the prospect of credits or a certificate, because he/she thinks that the more students attend such courses and obtain such certificates, the less they are worth. On the other hand, when a student really enjoys an MOOC course, his/her intention to learn with MOOCs increases as the force of subjective norms increases. 

### 4.1. Theoretical Implications

The findings of this study offer several implications for the extant knowledge. First, this study contributes to the understanding of motivation. The MOOC, an innovative educational model, has been widely studied in recent years from various aspects, including teachers’ experience sharing [63], MOOC technology introduction [64,65], and student attitudes to MOOCs [66,67]. However, few studies have explored a comprehensive framework of how students’ motivations affect their behavioral intentions. This study represents an initial attempt to explore whether students’ intention to learn with MOOCs is significantly affected by extrinsic and intrinsic motivations. 

Second, researchers have argued that mediator factor identification is especially valuable to better theoretical understanding [68]. This study extends our understanding of student MOOC learning by exploring an interesting mediator variable—technology fit. As an indispensable part of online education, previous studies on technology fit primarily focused on the construction of support platforms and systems, few studies focused specifically on the relationship between learning support services and learning intention from the perspective of students’ experience. Our research helps researchers better comprehend the underlying mechanism of the impact of motivation on student MOOC learning intention. 

Third, the successful identification of moderator variables is another important contribution of this study. As motivation is a complex construct and the MOOC context and content can alter or trigger changes in motivational states, we posit that subjective norms can have a positive influence on the relationship between extrinsic motivation and intention to learn with MOOCs and a negative influence on the relationship between intrinsic motivation and behavioral intention. However, the interesting findings uncovered by this study (i.e., subjective norms’ negative influence on the relationship between extrinsic motivation and behavioral intention and their positive influence on the relationship between intrinsic motivation and behavioral intention) extend the literature. In particular, this study is one of the first attempts to examine the moderation effect of subjective norms on the relationship between motivation and behavioral intention based on the extant understanding of the influence of subjective norms [69]. More importantly, the different mechanisms of subjective norms’ effect on intrinsic and extrinsic motivations were explored for the first time, which contributes significantly to knowledge. 

### 4.2. Practical Implications

The findings of this study also provide practical guidelines for developers of MOOC platforms and teachers. As suggested by the findings regarding the main and moderation effects, it is even more important for teaching practitioners to consider how to boost students’ intrinsic motivation to learn with MOOCs by means other than external rewards such as credits. Regarding the types of strategies practitioners should adopt in order to improve students’ intrinsic motivation, we think it is better to advertise the interestingness and value of various MOOCs. Meanwhile, MOOC platforms should, if possible, trace students’ learning and usage data, as well as their social network-related data, in order to personalize promotion strategies [70]. The finding regarding the mediation effect offers practical guidelines to practitioners on how to improve the fit between courses and MOOC learning, thus enhancing students’ evaluation of technology fit. To this end, it is also essential for practitioners to consider the types of courses that could be best provided through MOOCs, especially for those students who are high in extrinsic motivation but low in intrinsic motivation. 

### 4.3. Limitations and Research Directions

Despite the interesting findings and contributions, as with most research, this work has its limitations, which provide opportunities for future research. First, although university students are appropriate subjects for the research objective, the results are not ideal from the perspective of generalizability, which may limit readers’ understanding of the behavior of broader samples. Meanwhile, despite the fact that we used more than 200 student samples to test our model, which perfectly meets the requirement for statistics, considering that the large market of MOOCs in universities, the results are still, to some extent, limited by the sample size. Larger samples collected from more universities would be better. Further research is needed.

Second, despite the current debate in the literature regarding the interactive relationship between extrinsic motivation and intrinsic motivation, some studies have argued that extrinsic motivation can significantly improve people’s intrinsic motivation, while others have demonstrated that extrinsic motivation undermines the effect of intrinsic motivation [33,34], this study did not consider the possible effect of extrinsic motivation on intrinsic motivation. Thus, we call for further research to consider these interactive relationships. 

Third, this study examined the concept of perceived technology fit at a relatively abstract level. That is, we investigated students’ own evaluation of whether MOOCs provide a good way of learning, but we did not examine it in detail. Although this is reasonable and in line with previous studies [42], there are further interesting questions that still need in-depth investigation. For instance, can different types of MOOCs be evaluated differently? We also call for future research to explore this area.

## Figures and Tables

**Table 1:** Measurement items.

**Construct**	**Item**	**Source**
Intention to learn with MOOCs	(1) “What is the likelihood that you would learn from an MOOC?” (2) “How likely are you to learn with MOOCs?” (3) “I think I will learn with MOOCs after class.”	[53]
Intrinsic motivation	(1) “I will learn with MOOCs if it is interesting.” (2) “I will learn with MOOCs if it is fun.” (3) “I will learn with MOOCs if it is enjoyable.”	[54]
Extrinsic motivation	(1) “I will learn with MOOCs if it can improve my learning effectiveness.” (2) “I will learn with MOOCs if it can improve the quality of my work.” (3) “I will learn with MOOCs if it can improve my learning performance.” (4) “I will learn with MOOCs if it is useful for my learning.”	[55]
Perceived technology fit	(1) “MOOCs fit well with the way I like to study.” (2) “MOOCs are compatible with all aspects of my study.” (3) “It is easy to get MOOCs to do what I want them to do.”	[37]
Subjective norms	(1) “I would consider learning with MOOCs if my friends thought I should use it.” (2) “I would consider learning with MOOCs if my teachers thought I should use it.” (3) “I would consider learning with MOOCs if my classmates thought I should use it.”	[46]

**Table 2:** Factor loading analysis.

	Extrinsic Motivation (EM)	Intrinsic Motivation (IM)	Perceived Technology Fit (PTF)	Subjective Norm (SN)	Intention to Learn with MOOCs (ItoL)
EM 1	**0.817**	0.151	0.227	0.202	0.353
EM 2	**0.821**	0.164	0.286	0.224	0.336
EM 3	**0.820**	0.170	0.281	0.203	0.330
EM 4	**0.657**	0.219	0.376	0.271	0.329
PTF 1	0.362	0.118	**0.753**	0.176	0.099
PTF 2	0.540	0.120	**0.671**	0.118	0.241
PTF 3	0.130	0.114	**0.837**	0.307	0.196
PTF 4	0.232	0.130	**0.813**	0.310	0.228
ItoL 1	0.386	0.215	0.185	0.176	**0.803**
ItoL 2	0.352	0.167	0.212	0.182	**0.844**
ItoL 3	0.375	0.208	0.259	0.238	**0.744**
IM 1	0.089	**0.812**	0.175	0.270	0.029
IM 2	0.112	**0.875**	0.163	0.163	0.162
IM 3	0.216	**0.822**	–0.005	0.070	0.260
SN 1	0.365	0.221	0.268	**0.665**	0.294
SN 2	0.171	0.232	0.399	**0.755**	0.110
SN 3	0.252	0.284	0.284	**0.696**	0.272

**Table 3:** Mediating effects testing.

Path	β	*p*-Value
Path A: EM→ItoL	0.496	*p* < 0.001
Path A: IM→ItoL	0.289	*p* < 0.001
Path B: PTF→ItoL	0.664	*p* < 0.001
Path C: EM→PTF	0.649	*p* < 0.001
Path C: IM→PTF	0.209	*p* < 0.001
Path D: EM→PTF	0.646	*p* < 0.001
Path D: EM→ItoL	0.066	*p* = 0.295
Path D: IM→PTF	0.211	*p* < 0.001
Path D: IM→ItoL	0.148	*p* = 0.003

## Data Availability

Data supporting reported results are available from the authors on request.
